# Estimating infectiousness throughout SARS-CoV-2 infection course

**DOI:** 10.1126/science.abi5273

**Published:** 2021-05-25

**Authors:** Terry C. Jones, Guido Biele, Barbara Mühlemann, Talitha Veith, Julia Schneider, Jörn Beheim-Schwarzbach, Tobias Bleicker, Julia Tesch, Marie Luisa Schmidt, Leif Erik Sander, Florian Kurth, Peter Menzel, Rolf Schwarzer, Marta Zuchowski, Jörg Hofmann, Andi Krumbholz, Angela Stein, Anke Edelmann, Victor Max Corman, Christian Drosten

**Affiliations:** 1 Institute of Virology, Charité­–Universitätsmedizin Berlin, corporate member of Freie Universität Berlin, Humboldt-Universität zu Berlin, and Berlin Institute of Health, 10117 Berlin, Germany.; 2 German Centre for Infection Research (DZIF), partner site Charité, 10117 Berlin, Germany.; 3 Centre for Pathogen Evolution, Department of Zoology, University of Cambridge, Cambridge CB2 3EJ, U.K.; 4 Norwegian Institute of Public Health, 0473 Oslo, Norway.; 5 University of Oslo, 0315 Oslo, Norway.; 6 Department of Infectious Diseases and Respiratory Medicine, Charité–Universitätsmedizin Berlin, corporate member of Freie Universität Berlin and Humboldt-Universität zu Berlin, 10117 Berlin, Germany.; 7 Department of Tropical Medicine, Bernhard Nocht Institute for Tropical Medicine, and Department of Medicine I, University Medical Centre Hamburg-Eppendorf, 20359 Hamburg, Germany.; 8 Labor Berlin–Charité Vivantes GmbH, Sylter Straße 2, 13353 Berlin, Germany.; 9 Institute for Infection Medicine, Christian-Albrechts-Universität zu Kiel and University Medical Center Schleswig-Holstein, Campus Kiel, 24105 Kiel, Germany.; 10 Labor Dr. Krause und Kollegen MVZ GmbH, 24106 Kiel, Germany.

## Abstract

Two elementary parameters for quantifying viral infection and shedding are viral load and whether samples yield a replicating virus isolate in cell culture. We examined 25,381 cases of severe acute respiratory syndrome coronavirus 2 (SARS-CoV-2) in Germany, including 6110 from test centers attended by presymptomatic, asymptomatic, and mildly symptomatic (PAMS) subjects, 9519 who were hospitalized, and 1533 B.1.1.7 lineage infections. The viral load of the youngest subjects was lower than that of the older subjects by 0.5 (or fewer) log_10_ units, and they displayed an estimated ~78% of the peak cell culture replication probability; in part this was due to smaller swab sizes and unlikely to be clinically relevant. Viral loads above 10^9^ copies per swab were found in 8% of subjects, one-third of whom were PAMS, with a mean age of 37.6 years. We estimate 4.3 days from onset of shedding to peak viral load (10^8.1^ RNA copies per swab) and peak cell culture isolation probability (0.75). B.1.1.7 subjects had mean log_10_ viral load 1.05 higher than that of non-B.1.1.7 subjects, and the estimated cell culture replication probability of B.1.1.7 subjects was higher by a factor of 2.6.

## Correlates of infectiousness

The role that individuals with asymptomatic or mildly symptomatic severe acute respiratory syndrome coronavirus 2 have in transmission of the virus is not well understood. Jones *et al.* investigated viral load in patients, comparing those showing few, if any, symptoms with hospitalized cases. Approximately 400,000 individuals, mostly from Berlin, were tested from February 2020 to March 2021 and about 6% tested positive. Of the 25,381 positive subjects, about 8% showed very high viral loads. People became infectious within 2 days of infection, and in hospitalized individuals, about 4 days elapsed from the start of virus shedding to the time of peak viral load, which occurred 1 to 3 days before the onset of symptoms. Overall, viral load was highly variable, but was about 10-fold higher in persons infected with the B.1.1.7 variant. Children had slightly lower viral loads than adults, although this difference may not be clinically significant.

*Science*, abi5273, this issue p. eabi5273

## Abstract (structured)

**INTRODUCTION:**

Although post facto studies have revealed the importance of severe acute respiratory syndrome coronavirus 2 (SARS-CoV-2) transmission from presymptomatic, asymptomatic, and mildly symptomatic (PAMS) cases, the virological basis of their infectiousness remains largely unquantified. The reasons for the rapid spread of variant lineages of concern, such as B.1.1.7, have yet to be fully determined.

**RATIONALE:**

Viral load (viral RNA concentration) in patient samples and the rate of isolation success of virus from clinical specimens in cell culture are the clinical parameters most directly relevant to infectiousness and hence to transmission. To increase our understanding of the infectiousness of SARS-CoV-2, especially in PAMS cases and those infected with the B.1.1.7 variant, we analyzed viral load data from 25,381 German cases, including 9519 hospitalized patients, 6110 PAMS cases from walk-in test centers, 1533 B.1.1.7 variant infections, and the viral load time series of 4434 (mainly hospitalized) patients. Viral load results were then combined with estimated cell culture isolation probabilities, producing a clinical proxy estimate of infectiousness.

**RESULTS:**

PAMS subjects had, at the first positive test, viral loads and estimated infectiousness only slightly less than hospitalized patients. Similarly, children were found to have mean viral loads only slightly lower (0.5 log_10_ units or less) than those of adults and ~78% of the adult peak cell culture isolation probability. Eight percent of first-positive viral loads were 10^9^ copies per swab or higher, across a wide age range (mean 37.6 years, standard deviation 13.4 years), representing a likely highly infectious minority, one-third of whom were PAMS. Relative to non-B.1.1.7 cases, patients with the B.1.1.7 variant had viral loads that were higher by a factor of 10 and estimated cell culture infectivity that was higher by a factor of 2.6. Similar ranges of viral loads from B.1.1.7 and B.1.177 samples were shown to be capable of causing infection in Caco-2 cell culture. A time-course analysis estimates that a peak viral load of 10^8.1^ copies per swab is reached 4.3 days after onset of shedding and shows that, across the course of infection, hospitalized patients have slightly higher viral loads than nonhospitalized cases, who in turn have viral loads slightly higher than PAMS cases. Higher viral loads are observed in first-positive tests of PAMS subjects, likely as a result of systematic earlier testing. Mean culture isolation probability declines to 0.5 at 5 days after peak viral load and to 0.3 at 10 days after peak viral load. We estimate a rate of viral load decline of 0.17 log_10_ units per day, which, combined with reported estimates of incubation time and time to loss of successful cell culture isolation, suggests that viral load peaks 1 to 3 days before onset of symptoms (in symptomatic cases).

**CONCLUSION:**

PAMS subjects who test positive at walk-in test centers can be expected to be approximately as infectious as hospitalized patients. The level of expected infectious viral shedding of PAMS people is of high importance because they are circulating in the community at the time of detection of infection. Although viral load and cell culture infectivity cannot be translated directly to transmission probability, it is likely that the rapid spread of the B.1.1.7 variant is partly attributable to higher viral load in these cases. Easily measured virological parameters can be used, for example, to estimate transmission risk from different groups (by age, gender, clinical status, etc.), to quantify variance, to show differences in virus variants, to highlight and quantify overdispersion, and to inform quarantine, containment, and elimination strategies.

## Teaser

Analysis of thousands of people who tested positive in Germany reveals that many were asymptomatic and a minority exhibited high viral loads.

## Graphical abstract

**Figure:**
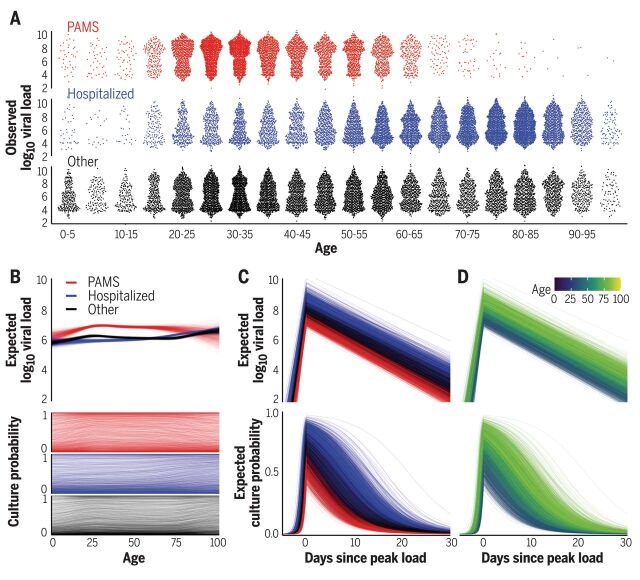
Viral load and cell culture infectivity in 25,381 SARS-CoV-2 infections. (**A**) Viral loads in presymptomatic, asymptomatic, and mildly symptomatic cases (PAMS; red), hospitalized patients (blue), and other subjects (black). (**B**) Expected first-positive viral load and cell culture isolation probability, colored as in (A). (**C**) Temporal estimation with lines representing patients, colored as in (A). (**D**) As in (C), but colored by age.

Respiratory disease transmission is highly context-dependent and difficult to quantify or predict at the individual level. This is especially the case when transmission from presymptomatic, asymptomatic, and mildly symptomatic (PAMS) subjects is frequent, as with severe acute respiratory syndrome coronavirus 2 (SARS-CoV-2) (*1*–*8*). Transmission is therefore typically inferred from population-level information and summarized as a single overall average, known as the basic reproductive number, R_0_. Although R_0_ is an essential and critical parameter for understanding and managing population-level disease dynamics, it is a resultant, downstream characterization of transmission. With regard to SARS-CoV-2, many finer-grained upstream questions regarding infectiousness remain unresolved or unaddressed. Three categories of uncertainty are (i) differences in infectiousness among individuals or groups such as PAMS subjects, according to age, gender, vaccination status, etc.; (ii) timing and degree of peak infectiousness, timing of loss of infectiousness, rates of infectiousness increase and decrease, and how these relate to onset of symptoms (when present); and (iii) differences in infectiousness due to inherent properties of virus variants.

These interrelated issues can all be addressed through the combined study of two clinical virological parameters: the viral load (viral RNA concentration) in patient samples, and virus isolation success in cell culture trials. Viral load and cell culture infectivity cannot be translated directly to in vivo infectiousness, and the impact of social context and behavior on transmission is very high; nonetheless, these quantifiable parameters can generally be expected to be those most closely associated with transmission likelihood. A strong relationship between SARS-CoV-2 viral load and transmission has been reported (*9*), comparing favorably with the situation with influenza virus, where the association is less clear (*10*, *11*).

The emergence of more transmissible SARS-CoV-2 variants, such as the B.1.1.7 lineage (UK Variant of Concern 202012/01), emphasizes the importance of correlates of shedding and transmission. The scarcity of viral load data in people with recent variants, and in PAMS subjects of all ages (*12*), is a blind spot of key importance because many outbreaks have clearly been triggered and fueled by these subjects (*2*, *13*–*17*). Viral load data from PAMS cases are rarely available, greatly reducing the number of studies with information from both symptomatic and PAMS subjects and that span the course of infections (*12*, *18*). Making matters worse, it is not possible to place positive reverse transcription polymerase chain reaction (RT-PCR) results from asymptomatic subjects in time relative to a nonexistent day of symptom onset, so these cases cannot be included in studies focused on incubation period. Additionally, viral load time courses relative to the day of symptom onset rely on patient recall, a suboptimal measure that is subject to human error and that overlooks infections from presymptomatic or asymptomatic contacts (*12*). An alternative and more fundamental parameter, the day of peak viral load, can be estimated from dated viral load time-series data, drawn from the entire period of viral load rise and fall and the full range of symptomatic statuses.

To better understand SARS-CoV-2 infectiousness, we analyzed viral load, cell culture isolation, and genome sequencing data from a diagnostic laboratory in Berlin (Charité–Universitätsmedizin Berlin Institute of Virology and Labor Berlin). We first address a set of questions regarding infectiousness at the moment of disease detection, especially in PAMS subjects whose infections were detected at walk-in community test centers. Because these people are circulating in the general community before their infections are detected, and are healthy enough to present themselves at such centers, their prevalence and shedding are of key importance to the understanding and prevention of transmission. In addition to PAMS subjects, we consider the infectiousness suggested by first-positive tests from hospitalized patients, including differences according to age, virus variant, and gender. A further set of temporal questions are then addressed by studying how infectiousness changes during the infection course. Using viral load measurements from patients with at least three RT-PCR tests, we estimate the onset of infectious viral shedding, peak viral load, and the rates of viral load increase and decline. Knowledge of these parameters enables fundamental comparisons between groups of subjects and between virus strains, and highlights the misleading impression created by viral loads from first-positive RT-PCR tests if the time of testing in the infection course is not considered.

## Study composition

We examined 936,423 SARS-CoV-2 routine diagnostic RT-PCR results from 415,935 subjects aged 0 to 100 years from 24 February 2020 to 2 April 2021. Samples were collected at test centers and medical practices mostly in and around Berlin, Germany, and analyzed with LightCycler 480 and cobas 6800/8800 systems from Roche. Of all tested subjects, 25,381 (6.1%) had at least one positive RT-PCR test (Table 1). Positive subjects had a mean age of 51.7 years with high standard deviation (SD) of 22.7 years, and a mean of 4.5 RT-PCR tests (SD 5.7), of which 1.7 (SD 1.4) were positive. Of the positive subjects, 4344 had tests on at least 3 days (with at least two tests positive) and were included in a time-series analysis.

**Table 1:** Age stratification of first-positive RT-PCR tests and viral load for 25,381 positive cases. *N*, number of subjects with a positive test result; Pos. %, percentage of positive subjects; Load (SD), mean log_10_(viral load) and standard deviation; ≥3 tests, number of subjects with at least three RT-PCR test results, as used in the viral load time course analysis. Age ranges (in years) are open-closed intervals.

	**All cases**				**PAMS cases**			**Hospitalized cases**		
**Age**	***N***	**Pos. %**	**Load (SD)**	**≥3 tests**	***N***	**Pos. %**	**Load (SD)**	***N***	**Pos. %**	**Load (SD)**
0–5	330	1.8	5.9 (1.84)	16	36	5.1	6.6 (1.87)	32	0.9	5.6 (2.22)
5–10	185	1.8	6.0 (1.73)	12	39	6.2	6.1 (1.83)	18	1.4	5.8 (1.97)
10–15	227	2.2	6.0 (1.76)	8	51	6.9	6.4 (1.92)	22	1.4	6.0 (2.02)
15–20	643	3.0	6.3 (1.87)	39	192	5.1	6.7 (1.77)	121	2.5	6.1 (1.95)
20–25	1637	3.2	6.5 (1.89)	110	696	4.0	6.9 (1.86)	246	2.7	5.9 (1.92)
25–35	4452	3.0	6.6 (1.90)	320	1988	3.9	7.0 (1.83)	614	2.2	6.0 (1.88)
35–45	3393	2.7	6.4 (1.84)	323	1277	3.5	6.9 (1.79)	576	2.0	6.0 (1.90)
45–55	3341	3.1	6.4 (1.81)	401	1012	3.4	6.9 (1.83)	733	2.3	5.9 (1.77)
55–65	3322	2.7	6.3 (1.78)	623	674	3.0	6.8 (1.82)	1039	2.1	5.9 (1.80)
>65	7851	3.0	6.4 (1.79)	2492	145	5.8	6.8 (1.87)	3434	2.3	6.2 (1.86)

**Fig. 1:**
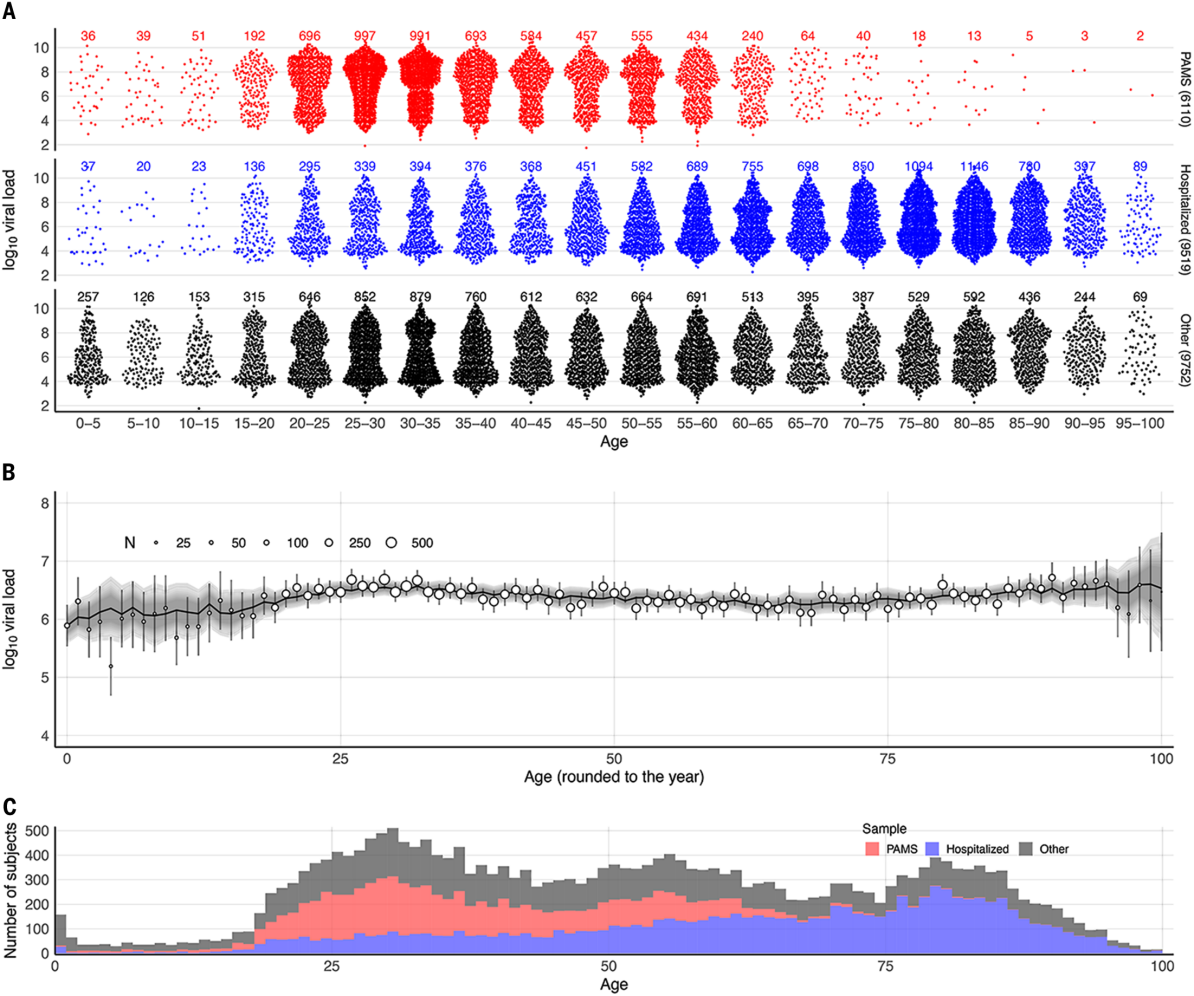
Distribution of age and first-positive viral load in PAMS, Hospitalized, and Other subjects. (**A**) Distribution of observed first-positive viral loads for 25,381 subjects according to clinical status (6110 PAMS, 9519 Hospitalized, 9752 Other) and age group. (**B**) Age–viral load association. Observed viral loads are shown as circles (circle size indicates subject count) with vertical lines denoting confidence intervals; model-predicted viral loads are shown as a black, roughly horizontal line, with gray shading denoting credible intervals. (**C**) Stacked age histograms according to subject clinical status. Because inclusion in the study required a positive RT-PCR test result, and because testing is in many cases symptom-dependent, the study may have a proportion of PAMS cases that differs from the proportion in the general population.

We divided the 25,381 positive subjects into three groups (Fig. 1). The Hospitalized group (9519 subjects, 37.5%) included all those who tested positive in an in-patient hospitalized context at any point in their infection. The PAMS group (6110 subjects, 24.1%) included people whose first positive sample was obtained in any of 24 Berlin COVID-19 walk-in community test centers, provided they were not in the Hospitalized category. The Other group (9752 subjects, 38.4%) included everyone not in the first two categories (table S1). As Fig. 1 shows, there were relatively low numbers of young subjects in all three groups, and very few elderly PAMS subjects. The validity of the PAMS classification is supported by the fact that of the overall 6159 infections detected at walk-in test centers, only 49 subjects (0.8%) were later hospitalized. Subjects testing positive at these centers are almost certainly receiving their first positive test because they are instructed to immediately self-isolate, and our data confirm that such subjects are rarely retested: Only 4.6% of people with at least three test results had their first test at a walk-in test center. Of the 9519 subjects who were ever hospitalized, 6835 were already in hospital at the time of their first positive test. PAMS subjects had a mean age of 38.0 years (SD 13.7), typically younger than Other subjects (mean 49.1 years, SD 23.5), with Hospitalized the oldest group (mean 63.2 years, SD 20.7). Typing RT-PCR indicated that 1533 subjects were infected with a strain belonging to the B.1.1.7 lineage, as confirmed by full genomes from next-generation sequencing (see materials and methods).

## First-positive viral load

Across all subjects, the mean viral load [given as log_10_(RNA copies per swab)] in the first positive-testing sample was 6.39 (SD 1.83). The PAMS subjects had viral loads higher than those of the Hospitalized subjects for ages up to 70 years, as exemplified by a 6.9 mean for PAMS compared to a 6.0 mean in Hospitalized adult subjects of 20 to 65 years. Crude comparisons of viral loads in age groups showed no substantial difference in first-positive viral load between groups of people older than 20 years (Table 1). Children and adolescents had mean first-positive viral load differences ranging between –0.49 (–0.69, –0.29) and –0.16 (–0.31, –0.01) relative to adults aged 20 to 65 (Table 2). Here and below, parameter differences between age groups show the younger value minus the older, so a negative difference indicates a lower value in the younger group. Ranges given in parentheses are 90% credible intervals.

**Table 2:** Pairwise age comparisons of first-positive RT-PCR viral load and estimated culture probability calculated from spline regression or raw data. Only the spline-based regression adjusts for effects of the test center and RT-PCR system. Differences are mean differences, with 90% credible intervals or confidence intervals from null-hypothesis significance testing given in parentheses. *P* values are from Mann-Whitney *U* tests (*96*).

		**Spline-based regression (adjusted)**		**Raw data (unadjusted)**	
**Sample**	**Comparison**	**Culture probability difference**	**log_10_(load difference)**	**log_10_(load difference)**	***P***
All	0–5 vs. 20–65	–0.067 (–0.167, –0.002)	–0.50 (–0.62, –0.37)	–0.49 (–0.69, –0.29)	<0.001
All	5–10 vs. 20–65	–0.054 (–0.132, –0.002)	–0.40 (–0.50, –0.30)	–0.38 (–0.64, –0.13)	0.004
All	10–15 vs. 20–65	–0.045 (–0.111, –0.002)	–0.30 (–0.39, –0.22)	–0.42 (–0.65, –0.18)	<0.001
All	15–20 vs. 20–65	–0.033 (–0.076, –0.001)	–0.18 (–0.23, –0.12)	–0.16 (–0.31, –0.01)	0.033
PAMS	0–5 vs. 20–65	–0.067 (–0.167, –0.002)	–0.50 (–0.62, –0.37)	–0.49 (–0.69, –0.29)	<0.001
PAMS	5–10 vs. 20–65	–0.112 (–0.279, –0.003)	–0.63 (–0.96, –0.32)	–0.37 (–1.00, 0.26)	0.213
PAMS	10–15 vs. 20–65	–0.092 (–0.228, –0.003)	–0.51 (–0.77, –0.26)	–0.86 (–1.46, –0.26)	0.004
PAMS	15–20 vs. 20–65	–0.064 (–0.162, –0.002)	–0.35 (–0.54, –0.17)	–0.56 (–1.10, –0.02)	0.034
Hospitalized	0–5 vs. 20–65	–0.033 (–0.087, –0.001)	–0.18 (–0.29, –0.07)	–0.26 (–0.52, –0.01)	0.046
Hospitalized	5–10 vs. 20–65	–0.028 (–0.104, 0.009)	–0.18 (–0.45, 0.07)	–0.36 (–1.10, 0.37)	0.115
Hospitalized	10–15 vs. 20–65	–0.025 (–0.084, 0.003)	–0.16 (–0.36, 0.03)	–0.48 (–1.38, 0.43)	0.172
Hospitalized	15–20 vs. 20–65	–0.022 (–0.071, 0.001)	–0.14 (–0.29, 0.02)	–0.11 (–0.97, 0.74)	0.625
Other	0–5 vs. 20–65	–0.018 (–0.055, 0.000)	–0.11 (–0.22, 0.01)	0.00 (–0.33, 0.33)	0.845
Other	5–10 vs. 20–65	–0.058 (–0.148, –0.001)	–0.36 (–0.51, –0.20)	–0.33 (–0.55, –0.10)	0.004
Other	10–15 vs. 20–65	–0.044 (–0.110, –0.001)	–0.27 (–0.39, –0.15)	–0.10 (–0.40, 0.20)	0.586
Other	15–20 vs. 20–65	–0.026 (–0.072, –0.001)	–0.16 (–0.27, –0.06)	–0.31 (–0.58, –0.04)	0.045

**Fig. 2:**
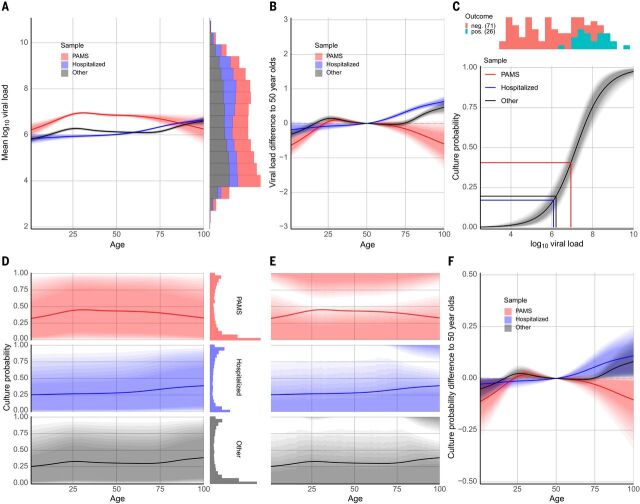
Estimated viral load and culture probability at time of first positive RT-PCR test. Shaded regions denote 90% credible intervals in all panels. To indicate change within each 90% region, shading decreases in intensity from a narrow 50% credibility interval level to the full 90%. (**A**) Estimated mean viral load in first-positive RT-PCR tests according to age and status. The stacked histogram (right) shows the observed viral load distribution. Because the shaded region shows the 90% credible interval for the mean, it does not include the higher values shown in the histogram on the right. (**B**) Differences in estimated first-positive viral load according to age and status. Each colored line is specific to a particular subset of subjects (PAMS, Hospitalized, Other). Each line shows how viral load differs by age for subjects of the corresponding status from that of 50-year-old (rounded age) subjects of the same status. The comparison against 50-year-olds avoids comparing any subset of the subjects against a value (such as the overall mean) that is computed in part on the basis of that subset, thereby partially comparing data to the same data. The mean first-positive viral loads for 50-year-old PAMS and Hospitalized subjects are 7.2 and 6.2, respectively, allowing relative *y*-axis differences to be translated to approximate viral loads. (**C**) Estimation of the association between viral load and cell culture isolation success rate based on data from our own laboratory (*19*) and Perera *et al*. (*20*). Viral load differences in the log_10_ range ~6 to ~9 have a large impact on culture probability, whereas the impact is negligible for differences outside that range. The vertical lines indicate the observed mean first-positive viral loads for different subject groups; the horizontal lines show the corresponding expected probabilities of a positive culture. (**D**) Estimated culture probability at time of first-positive RT-PCR according to age and status, obtained by combining the results in (A) and (C). Culture probability is calculated from posterior predictions [i.e., the posterior means shown in (A) plus error variance]. The histogram at right shows that mean culture probabilities calculated from observed viral loads are not well matched by credible intervals, which do not include the most probable estimated culture probabilities. (**E**) Culture probability with highest–posterior density regions, which do include the most probable estimated culture probabilities and match the histograms in (D) well. The *y* axis is the same as in (D). (**F**) Differences of estimated expected culture probability at time of first-positive RT-PCR for age groups, with plot elements as described for (B).

We used a Bayesian thin-plate spline regression to estimate the relationship among age, clinical status, and viral load from the first positive RT-PCR of each subject, adjusting for gender, type of test center, and PCR system used. The Bayesian model well represents the observed data (Fig. 1B, Table 2, and fig. S1). The raw data and the Bayesian estimation (Fig. 2A) suggest consideration of subjects in three age categories: young (ages 0 to 20 years, grouped into 5-year brackets), adult (20 to 65 years), and elderly (over 65 years). We estimated an average first-positive viral load of 6.40 (6.37, 6.42) for adults and a similar mean of 6.35 (6.32, 6.39) for the elderly (Fig. 2A). Younger age groups had lower mean viral loads than adults, with the difference falling steadily from –0.50 (–0.62, –0.37) for the very youngest (0 to 5 years) to –0.18 (–0.23, –0.12) for older adolescents (15 to 20 years) (Table 2). Young age groups of PAMS subjects had lower estimated viral loads than older PAMS subjects, with differences ranging from –0.18 (–0.29, –0.07) to –0.63 (–0.96, –0.32). Among Hospitalized subjects these differences were smaller, ranging from –0.18 (–0.45, 0.07) to –0.11 (–0.22, 0.01) (Table 2 and Fig. 2B). Viral loads of subjects younger than 65 years were ~0.75 higher for PAMS subjects than for Hospitalized subjects (Fig. 2A), likely because of a systematic difference in RT-PCR test timing, discussed below.

## Associating viral load with cell culture infectivity

We estimated the association between viral load and successful cell culture isolation probability (hereafter “culture probability”) by combining the viral load estimated from the Bayesian regression with cell culture isolation data from our own laboratory (*19*) and from Perera *et al*. (*20*) (Fig. 2C). Across all ages, the average estimated culture probability at the time of first positive RT-PCR was 0.35 (0.01, 0.94). The mean culture probability for PAMS cases, 0.44 (0.01, 0.98), was higher than for Hospitalized cases, 0.32 (0.00, 0.92) (Fig. 2D). Comparing PAMS cases, we found differences, in particular for children aged 0 to 5 compared to adults aged 20 to 65, with average culture probabilities of 0.329 (0.003, 0.950) and 0.441 (0.008, 0.981) respectively, and a difference of –0.112 (–0.279, –0.003). Age group differences in Hospitalized cases ranged from –0.028 (–0.104, 0.009) to –0.018 (–0.055, 0) (Table 2).

First-positive viral loads are weakly bimodally distributed (Figs. 1A and 2A), which is not reflected in age-specific means. The resultant distribution includes a majority of subjects with relatively low culture probability and a minority with very high culture probability (Fig. 2E and fig. S2). The highly infectious subset includes 2228 of 25,381 positive subjects (8.78%) with a first-positive viral load of at least 9.0, corresponding to an estimated culture probability of ~0.92 to 1.0. Of these 2228 subjects, 804 (36.09%) were PAMS at the time of testing, with a mean (median) age of 37.6 (34.0) and SD of 13.4 years. PAMS subjects are overrepresented in this highly infectious group among people aged 20 to 80 years, and Hospitalized subjects are overrepresented in people aged 80 to 100 years (fig. S3).

## Estimating B.1.1.7 infectiousness at first-positive test

**Fig. 3:**
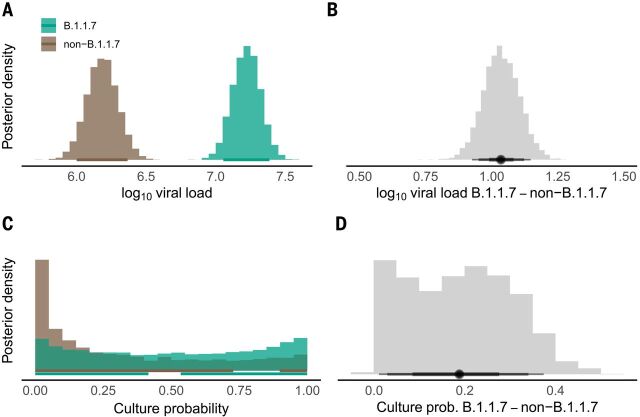
Posterior distributions of estimated viral loads and culture probabilities for B.1.1.7 and non-B.1.1.7 subjects, and their differences. Viral loads and estimated culture probabilities of 1387 B.1.1.7 subjects and 977 non-B.1.1.7 subjects are represented. To select a comparable subset of non-B.1.1.7 viral loads for the comparison, we included only non-B.1.1.7 subjects from test centers that had detected a B.1.1.7 variant as well as at least one non-B.1.1.7 subject, and only if the non-B.1.1.7 infection was detected on the same day as a B.1.1.7 infection was detected, plus or minus 1 day. Similar differences exist when viral loads from larger, less restrictive, subsets of non-B.1.1.7 subjects are used in the comparison (table S2; see materials and methods). (**A**) Posterior distribution of viral load. (**B**) Posterior distribution of difference of average viral load between B.1.1.7 and non-B.1.1.7 cases. (**C**) Posterior distribution of the estimated culture probability. See also fig. S2. (**D**) Difference of mean culture probability between B.1.1.7 and non-B.1.1.7 cases. Horizontal lines indicate 90% credible intervals in (A), (B), and (D) and the highest posterior density intervals in (C).

The 1533 subjects infected with a B.1.1.7 virus in our dataset had an observed mean first-positive viral load of 7.38 (SD 1.54), which is 1.05 higher (0.97, 1.13) than non-B.1.1.7 subjects in the full dataset. To increase specificity, we compared 1453 B.1.1.7 cases with 977 non-B.1.1.7 cases using viral loads only from centers with B.1.1.7 and non-B.1.1.7 cases, and only from the same day or 1 day before or after the B.1.1.7 sample was taken. This analysis adjusted for clinical status, gender, RT-PCR system, and subject age, and also modeled random test center effects. The results show that B.1.1.7 cases are associated with a 1.0 (0.9, 1.1) higher viral load (Fig. 3 and table S2). This results in a mean estimated B.1.1.7 subject culture probability of 0.50 (0.03, 0.97), considerably higher than the overall figure of 0.31 (0.00, 0.94) for the non-B.1.1.7 subjects in the comparison, corresponding to a median factor of 2.6 (50% credible interval: 1.4, 5.1) higher culture probability for samples from B.1.1.7 cases. To investigate whether there might be a difference in cell culture infectivity due to a factor other than viral load, we isolated virus from 105 samples (22 B.1.1.7, 83 B.1.177) in Caco-2 cells from a collection of 223 samples with matched viral loads. Although no statistical difference was seen in the distribution of viral loads that resulted in successful isolation (fig. S4), uncertainty attributable to the routine diagnostic laboratory context—including uncontrolled preanalytical parameters such as transportation time and temperature, together with the small isolation-positive sample sizes—are insufficient to support a conclusion that the distributions do not differ (see materials and methods).

## Estimating infectiousness over time

**Fig. 4:**
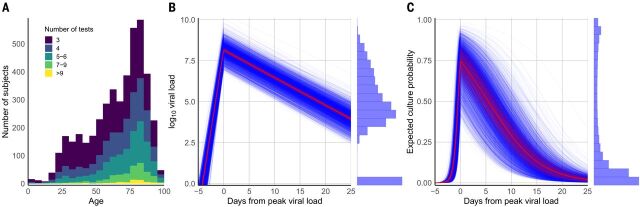
Viral load and estimated infectious virus shedding time series. Of 25,381 positive subjects, 4344 had three or more RT-PCR test results available, and these were used in a viral load time-series analysis. Subjects with only one result cannot be placed in time because of inherent ambiguity (given that the model has both an increasing and a decreasing phase), and those with only two test results are excluded from the time-series analysis because of insufficient data for temporal placement (their number of data points is less than the number of model parameters being estimated). (**A**) Number of subjects with three or more RT-PCR test results available, at least two of which were positive, according to age. (**B**) Estimated time course of viral load for 18,136 RT-PCR results from the 4344 subjects with at least three RT-PCR results. Blue lines are expected complete time courses for individual cases. The sample mean is shown in red, with its 90% credible interval as a shaded area. The histogram at right shows the distribution of all observed viral loads. The histogram values at zero correspond to the initial and trailing negative tests in subject timelines. Figure S8 shows raw viral load time series, per subject and split by number of RT-PCR tests. (**C**) Estimated time course of positive cell culture probability, calculated by applying the results shown in Fig. 2C to the estimated viral load time courses in (B). Blue lines are expected time courses for individual subjects. The sample average is shown in red, with its 90% credible interval as a shaded area. The histogram at right shows the distribution of culture probabilities in the sample and was obtained by applying the curve in Fig. 2C to the data in the histogram in (B).

To investigate viral load over the course of the infection, we estimated the slopes of a model of linear increase and then decline of log_10_ viral load using a Bayesian hierarchical model. The analysis used the time series of the 4344 subjects who had RT-PCR results on at least 3 days (with at least two tests being positive). The number of subjects with multiple test results skews heavily toward older subjects, with very few below the age of 20 meeting the criterion (Fig. 4A). We estimated time from onset of shedding to peak viral load of 4.31 (4.04, 4.60) days, mean peak viral load of 8.1 (8.0, 8.3), and mean decreasing viral load slope of –0.168 (–0.171, –0.165) per day (fig. S5). Figure S6 shows that while Hospitalized patients are estimated to be uniformly highly infectious at peak viral load, the infectiousness of PAMS subjects at peak load is more variable.

**Fig. 5:**
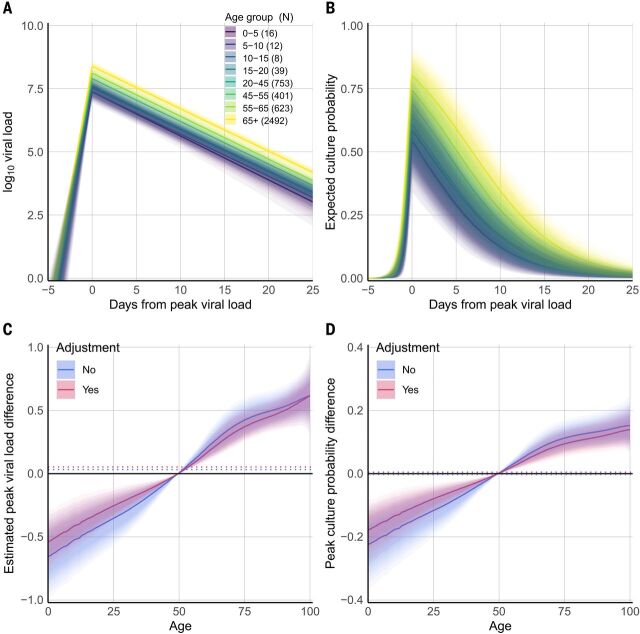
Estimated expected viral load and culture probability for age groups by time. (**A**) Change in estimated viral load over time according to age group for 4344 subjects with at least three RT-PCR tests, at least two of which were positive. Shading indicates the 90% credible interval of the mean. (**B**) Change in estimated culture probability over time according to age. Age groups, coloring, and shading are as in (A). (**C**) Estimated age group differences in mean peak viral load, corresponding to the values at day zero in (A). (**D**) Estimated age group differences in mean peak culture probability, corresponding to the values at day zero in (B). In (C) and (D), adjusted differences account for variations by age in clinical status and gender. Dotted lines indicate grand means for the 4344 subjects.

The temporal placement of the full 18,136 RT-PCR results from these 4344 subjects (80% of whom were hospitalized with COVID-19 at some point in their infections) is shown in fig. S7. Per-subject trajectories can differ considerably from that described by the mean parameters (Fig. 4B and fig. S8). Across all subjects, PAMS cases were on average detected 5.1 (4.5, 5.7) days after peak load, 2.4 (1.7, 3.0) days before non-PAMS cases, which were on average detected 7.4 (7.2, 7.6) days after peak load. We estimate that 962 (914, 1010) of the 4344 subjects [22.14% (21.04, 23.25)] had a first positive test before the time of their peak viral load, with a mean of 1.4 (1.3, 1.5) days before reaching peak viral load. Among the infections detected after peak viral load, the timing of the first positive RT-PCR test is estimated at 9.8 (9.6, 10.0) days after peak viral load, with SD of 6.9 (6.8, 7.0) days, reflecting a broad time range of infection detection. Estimated peak viral loads were higher in Hospitalized subjects than in Other subjects, and higher in Other subjects than in PAMS subjects, with differences of 0.68 (0.83, 0.52) and 0.96 (0.33, 1.53) respectively (fig. S9 and table S3). No differences according to gender were seen. Viral load time courses were similar across age groups, although younger subjects had lower peak viral load than adults aged 45 to 55 (Fig. 5, A and C, fig. S10, and table S4). Model parameters suggest a slightly longer time to peak, a higher peak, and a more rapid decline in viral load when the analysis is restricted to subjects with successively higher numbers of RT-PCR results (fig. S11 and table S5), with an increasing percentage of hospitalized subjects. Differences in model parameters according to the number of tests in subjects may reflect increased parameter accuracy due to additional data, although other factors associated with being tested more frequently may be responsible. The Bayesian estimation of the model agrees well with a separate second implementation based on simulated annealing (fig. S12, table S5, and supplementary text).

We estimate that the rise from near-zero to peak culture probability takes 1.8 (1.3, 2.6) days, with a mean peak culture probability of 0.74 (0.61, 0.85). Mean culture probability then declines to 0.52 (0.40, 0.64) at 5 days and to 0.29 (0.19, 0.40) at 10 days after peak viral load. Subject-level time courses can deviate substantially from these mean estimates (Fig. 4C). Peak culture probabilities for age groups range from a low of 0.54 (0.39, 0.71) for 0- to 5-year-olds to 0.80 (0.67, 0.90) for subjects more than 65 years old. The least infectious youngest children have 78% (61, 94) of the peak culture probability of adults aged 45 to 55 (Fig. 5, B and D, and table S4). An insufficient amount of data precludes a reliable B.1.1.7 viral load time-series analysis at this point.

## Discussion

### Limitations

Our analysis attempted to account for the effects of gender, PCR system, and test center type. Although we could not incorporate inter-run variability or the variability in the sample preanalytic (such as type of swab or initial sample volume) in our conversion of RT-PCR cycle threshold values to log_10_(viral load) values, these variabilities apply to all age groups and do not affect the interpretation of data for the purpose of our study. If the proportion of subjects with a certain clinical status differs between age groups in the study sample, this could lead to over- or underestimation of differences in viral load between age groups. However, as our study compares viral load between age groups stratified by clinical status, it appears unlikely that differential testing biases our results.

### Interpreting first-positive viral loads

Viral loads and their differences are not easy to interpret without knowledge of when in the disease course the samples were taken, and of the correspondence between viral load and shedding. The higher first-positive viral loads in PAMS subjects than in Hospitalized subjects are likely due to time of detection. This is suggested in the first place by the estimated difference of 2.4 (1.7, 3.0) days in test timing, which would produce a viral load difference of ~0.4 using the –0.168 daily viral load decline gradient from the (mainly hospitalized) time-series subjects. Additionally, from the time series of PAMS, Other, and Hospitalized subjects, we can estimate that throughout the infection course, the Hospitalized group has higher viral loads than the Other group, whose viral loads are in turn higher than those of the PAMS group (fig. S9 and table S3). This relationship holds across age groups (fig. S13) and also in a fine-grained split of test centers by clinical severity (fig. S14). Similarly, the lower first-positive viral loads in elderly PAMS subjects may be due to these subjects being less likely to be tested as early because they are more likely to be house-bound, less likely to be employed, less mobile, more cautious (therefore disinclined to get tested with only mild symptoms), etc. The impact on infectiousness of differences in viral load must be informed by where the viral loads fall on the viral load–culture probability curve. In our data, the viral loads involved in the difference between means in children and adults and the difference between means in B.1.1.7 and non-B.1.1.7 subjects result in quite different corresponding culture probabilities (see below).

### A highly infectious minority and overdispersion

The bimodal distribution of culture probabilities (Fig. 2, D and E) shows a small group of 8.78% of highly infectious subjects. This qualitatively agrees with a model (*21*) and a study (*22*) concluding that 10% and 15% of index cases, respectively, may be responsible for 80% of transmission. Other studies reported that 8 to 9% of individuals harbored 90% of total viral load (*23*), and that in cases from India (*24*) and Hong Kong (*6*) ~70% of index cases had no secondary cases. PAMS subjects can be construed to pose a risk for several reasons: 36.1% of the highly infectious subjects in our study were PAMS at the time of the detection of their infection, their mean age was 37.6 years with a high standard deviation of 13.4 years (figs. S2 and S3), and we estimate that infectiousness peaks 1 to 3 days before onset of symptoms (if any).

### Comparison with influenza virus

Without direct knowledge from a large number of SARS-CoV-2 transmission events, we could try to draw conclusions regarding infectiousness from studies of other respiratory viruses, such as influenza. However, it has become clear that there are important differences and uncertainties that would cast doubt on such a comparison. Influenza may have later onset of viral shedding; shedding finishes earlier; there may be a lower secondary attack rate; viral loads are much lower; there is variation between virus subtypes; the role of asymptomatic subjects in transmission is uncertain or thought to be reduced; and the frequency of asymptomatic infections is uncertain, especially in children (*10*, *11*, *25*–*29*). Age-specific behavioral differences do, however, make a large contribution to the established higher shedding of children relative to adults in influenza. This should be an important consideration for SARS-CoV-2, as shown by studies indicating higher transmission between children of similar ages (*6*, *24*) and high transmission heterogeneity (*22*). Despite many decades of close study of influenza virus, the relationship between viral load and transmission is unclear (*10*, *11*). The situation with respiratory syncytial virus is even less clear (*30*). Understanding SARS-CoV-2 transmission will likely be at least as challenging, given the high frequency of transmission from PAMS subjects (*1*–*8*). This suggests an important role for clinical parameters, given the apparently strong association between viral load and transmission, independent of symptoms (*9*).

### Estimated infectiousness in the young

The differences we observe in first-positive RT-PCR viral load between groups based on age are minor, as in other studies (*31*–*35*), and the viral loads in question—in the range of 5.9 to 6.6 (Table 1)—are in a region of the viral load–culture probability association where changes in viral load have relatively little impact on estimated culture probability (Fig. 2C). Comparisons between adult viral loads and those of children, and the relative infectious risks they pose, are impeded by the likely influence of nonviral factors. Nasopharyngeal swab samples, which often carry higher viral loads, are rarely taken from young children because they can be painful, and the sample volume carried by smaller pediatric swab devices is lower than in larger swabs used for adults (*36*). Infections in mildly symptomatic children may be initially missed and only detected later (*37*), resulting in lower first-positive viral loads. Our results of similar viral load trajectories for children and adults (Fig. 5), and the numeric range of the viral load values in question (Fig. 2C), suggest that viral load differences between children and adults are too small to be solely responsible for large differences in infectiousness. The impact on transmission of general age-related physiological differences, such as different innate immune responses (*38*), may be small relative to the impact of large differences in frequency of close contacts and transmission opportunities.

### Timing of estimated peak infectiousness relative to onset of symptoms

We estimated the time from onset of shedding to peak viral load at 4.3 days. Previous studies and reviews of COVID-19 report mean incubation times of 4.8 to 6.7 days (*4*, *39*–*44*), which suggests that, on average, a period of high infectivity can start several days before the onset of symptoms. Viral load rise may vary between individuals, and limitations of the available data suggest that our analysis may underestimate interindividual variation in viral load increase. The failure to isolate virus in cell culture beyond 10 days from symptom onset (*19*, *20*, *35*, *45*, *46*), together with our estimated slope of viral load decline, also suggest that peak viral load occurs 1 to 3 days before symptom onset (supplementary text). Data from 171 hospitalized patients from a Charité-Universitätsmedizin cohort suggest a figure of 4.3 days (fig. S15 and supplementary text).

### Estimated infectiousness of the B.1.1.7 variant

We found that people infected with a B.1.1.7 virus had a first-positive viral load that was ~1 higher than in people infected with a wild-type virus. The scale of the viral load difference, and its presence in the comparison between B.1.1.7-infected and non–B.1.1.7-infected subjects drawn from the same test centers at the same times, argue that the difference is not due to a systematic difference in time of sampling. The higher B.1.1.7 viral load can be compared to the findings of two large and closely controlled UK studies, a mortality study (*47*) and a vaccine trial (*48*), which imply higher B.1.1.7 viral loads by a factor of 5 to 10 (based on RT-PCR cycle threshold differences of 2.3 and ~3, respectively). Several other studies also appear to point to a higher B.1.1.7 viral load (*49*–*52*) (supplementary text).

The mean B.1.1.7 viral load value in our study falls in a region of the viral load–culture probability curve with a steep gradient (Fig. 2C), resulting in an estimated culture probability considerably higher than for non-B.1.1.7 subjects. Although a strong correlation has been observed between SARS-CoV-2 viral load and transmission (*9*), here we are estimating infectivity probability from cell culture trials. Any impact of a change in viral load on transmission will be highly dependent on context, so the large difference in estimated culture probability in our data is only a proxy indication of potentially higher transmissibility of the B.1.1.7 strain. We estimate that B.1.1.7-infected subjects’ mean culture probability is higher than that of non–B.1.1.7-infected subjects by a factor of 2.6. This can be compared to a UK study that found a factor of 1.3 relative increase in secondary attack rates for B.1.1.7 index cases in ~60,000 household contacts (*53*), a UK study estimating a factor of 1.7 to 1.8 increase in transmission (*54*), and an estimate of a 43% to 90% higher reproductive number (*55*).

### Summary

Our results indicate that PAMS subjects in apparently healthy groups can be expected to be as infectious as hospitalized patients at the time of detection. The relative levels of expected infectious virus shedding of PAMS subjects (including children) is of high importance because these people are circulating in the community and it is clear that they can trigger and fuel outbreaks (*56*). The results from our time-series analysis, and their generally good agreement with results from studies based on other metrics (often epidemiological), show that accurate estimations can be directly obtained from two easily measured virological parameters, viral load and sample cell culture infectivity. Such results can be put to many uses: to estimate transmission risk from different groups (by age, gender, clinical status, etc.), to quantify variance, to show differences in virus variants, to highlight and quantify overdispersion, and to inform quarantine, containment, and elimination strategies. Our understanding of the timing and magnitude of change in viral load and infectiousness, including the impact of influencing factors, will continue to improve as data from large studies accumulate and are analyzed. A major ongoing challenge is to connect what we learn about estimated infectiousness from these clinical parameters to highly context-dependent in vivo transmission. On the basis of our estimates of infectiousness of PAMS subjects and the higher viral load found in subjects infected with the B.1.1.7 variant, we can safely assume that nonpharmaceutical interventions such as social distancing and mask wearing have been key in preventing many additional outbreaks. Such measures should be used in all social settings and across all age groups wherever the virus is present.

## Materials and methods

### Age ranges

Age categories for the analysis of the first-positive test results mentioned in the text indicate mathematically open-closed ranges of years (e.g., 0-5 signifies (0-5] years). We group subjects up to 20 years old into age categories spanning 5 years, subjects from 20 to 65 years into an adult group, and elderly subjects into a 65+ category. This categorization is motivated by the observed data and the Bayesian estimation of viral load differences between children of different ages and adults. The age groupings used in the viral load time-series analysis are broader in the younger categories to increase the cardinality of those groups, because few young people have at least three RT-PCR tests (Fig. 4A).

### Viral loads

Viral load is semiquantitative, estimating RNA copies per entire swab sample, whereas only a fraction of the volume can reach the test tube. The quantification is based on a standard preparation tested in multiple diluted replicates to generate a standard curve and derive a formula in which RT-PCR cycle threshold values are converted to viral loads. This approach does not reflect inter-run variability or the variability in the sample preanalytic, such as type of swab or initial sample volume (varying between 2.0 and 4.3 ml). However, these variabilities apply to all age groups and do not affect the interpretation of data for the purpose of the present study.

Viral load figures are given as the logarithm base 10. Viral load is estimated from the cycle threshold (Ct) value using the empirical formulae 14.159 – (Ct × 0.297) for the Roche Light Cycler 480 system and 15.043 – (Ct × 0.296) for the Roche cobas 6800/8800 systems. The formulae are derived from testing standard curves and cannot be transferred to calculate viral load in other laboratory settings. Calibration of the systems and chemistries in actual use is required.

### B.1.1.7 viral load analysis

No analysis regarding symptomatic status was made for B.1.1.7 subjects because of uncertainties regarding exact operational protocols at outbreak hospitals. B.1.1.7 assignment to samples was initially made according to typing RT-PCR tests that detect the N501Y and 69/70 deletion in the amino acid sequence of the virus spike protein. Examination of the complete viral genome of 49 samples confirmed that the subjects were in fact infected with the B.1.1.7 variant, with all variant-defining substitutions and deletions (*57*) found in all cases. No consistent additional mutations or deletions/insertions were found in the sequences.

Sequencing read mapping was performed with Bowtie, with alignment using MAFFT and visual inspection using Geneious Prime (all version numbers given below). For the statistical comparison of B.1.1.7 and non-B.1.1.7 subjects, we identified test centers (hospital departments or wards, or organizations outside hospitals) that reported B.1.1.7 cases, and chose as comparison groups non-B.1.1.7 cases that were detected in these test centers on the same day or 1 day earlier or later. By modeling random effects for test centers, we estimate the expected viral load difference as the average of the within-test center differences. The consistent effect of B.1.1.7 throughout a range of comparison scenarios is shown in table S2.

### Sample type

An estimated 3% of our samples were from the lower respiratory tract. These were not removed from the dataset because of their low frequency and the fact that the first samples for patients are almost universally swab samples. Samples from the lower respiratory tract are generally taken from patients only after intubation, by which point viral loads have typically fallen.

### PAMS status

Metadata needed to discriminate patients into subcohorts on the basis of underlying diseases, outcome, or indications for diagnostic test application, including symptomatic status, were not always available. In the absence of subject-level data, we inferred PAMS status using the type of submitting test center as an indicator, classifying subjects as PAMS at the time of testing if their first-positive sample was taken from a walk-in COVID-19 test center and the subject had no later RT-PCR test done in a hospitalized context (e.g., in a ward or an intensive care unit). The correspondence between viral load and PAMS status derived herein may therefore be less accurate than in studies with subject-level symptom data. However, we make no formal claims regarding symptomatic status, and instead emphasize the fact that these PAMS subjects were healthy enough to be presenting at walk-in COVID-19 test centers, and were therefore capable to some extent, at that time, of circulating in the general community.

### Bayesian analysis of age–viral load associations

We estimated associations of viral load and age with a thin-plate spline regression using the brms package (*58*, *59*) in R (*60*). Spline coefficients were allowed to vary between groups determined by the clinical status (PAMS, Hospitalized, or Other), and random intercepts captured effects of test centers. To reduce the impact of outliers, we used Student *t*–distributed error terms. The analysis additionally accounted for baseline differences between subject groups, B.1.1.7 status, gender, and for the effect of the RT-PCR system. We also estimated the association between viral load and culture probability in order to calculate the expected culture probability at different age levels. This analysis used weakly informative priors and was estimated using four chains with 1000 warm-up samples and 2000 post–warm-up samples. Convergence of MCMC chains was examined by checking that potential scale reduction factors (R-hat) values were below 1.1. All calculations of age averages and group differences are based on posterior predictions generated from estimated model parameters. Expected probabilities of positive cultures (and their differences) were calculated by applying the posterior distribution of model parameters from the culture probability model to posterior predictions from the age association model.

### Combining culture probability data

To estimate the association between viral load and culture probability, we used data previously described by Wölfel (*19*) and Perera (*20*). Four other datasets could not be included because Ct values were not converted to viral loads (*35*, *46*, *61*, *62*). The data from the study by van Kampen *et al*. (*63*) were not included because they differed (by viral load of ~1.0) from the data used for the current analysis (*97*); this is likely due to a combination of factors including many patients who were in critical or immunocompromised condition, a high proportion of samples obtained from the lower respiratory tract (including late in the infectious course), and likely differences in cell culture trials. It is unsurprising that these data result in a shifted viral load/culture probability curve, and we excluded them because our focus was largely on first positive RT-PCR results from the upper respiratory tract, including from many subjects who were PAMS. [See (*97*) for a figure comparing the plot of the van Kampen dataset to the two we used.] To calculate the expected culture probability, by age (as in Fig. 2D) or by day from peak viral load (as in Fig. 4C), we combined the estimated viral loads (Figs. 2A and 4B) with the results of the regression of culture probability shown in Fig. 2C. We used posterior predictions from the age regression model, which reflect the variation of viral load within age groups, to estimate culture probabilities by age. For instance, to obtain the culture probability for a specific age and group, we look up the estimated (expected) viral load for that group, add an error term according to the estimated error variance, and, using the association shown in Fig. 2C, determine the expected culture probability. We used expected time courses (i.e., the model’s best guess for a time course) to estimate culture probability time courses.

### B.1.1.7 isolation data

The Institute of Virology at Charité–Universitätsmedizin Berlin routinely receives SARS-CoV-2–positive samples for confirmatory testing and sequencing. For this study we used anonymized remainder samples from a large laboratory in northern Germany, which were all stored in phosphate-buffered saline (PBS) and therefore suitable for cell culture isolation trials. Sample transport to the originating lab and later to Berlin was unrefrigerated, via road. As part of the routine testing, these samples were classified by typing RT-PCR and complete genome sequencing (*64*); 113 B.1.1.7 lineage samples and 110 B.1.177 lineage samples were selected, with approximately matched (pre-inoculation) SARS-CoV-2 RNA concentrations. Caco-2 (human colon carcinoma) cell cultures (*65*) were inoculated twice from each sample, once with undiluted material and once with a 1:10 dilution. The diluted inoculant was used to reduce the probability of culturing failure due to the possible presence of host immune factors (antibodies, cytokines, etc.) that might have a negative impact on isolation success, and to reduce the possibility of other unrelated agents (bacteria, fungi, etc.) resulting in cytopathic effect in the culture system. For cell culture isolation trials, 1.6 × 10^5^ cells were seeded per well in a 24-well plate. Cells were inoculated with swab suspensions for 1 hour at 37°C, subsequently rinsed with PBS, and fed with 1 ml of fresh Dulbecco’s modified Eagle’s minimum essential medium (DMEM; ThermoFisher Scientific) supplemented with 2% fetal bovine serum (FBS; Gibco), penicillin and streptomycin (P/S; 100 U/ml and 100 μg/ml, respectively; ThermoFisher Scientific), and amphotericin B (2.5 μg/ml; Biomol), then incubated for 5 days before harvesting supernatant for RT-PCR testing. Positive cell culture isolation was defined by a minimum 10× higher SARS-CoV-2 RNA load in the supernatant compared to the inoculant and signs of a typical SARS-CoV-2 cytopathic effect. Culture isolation was successful for 22 B.1.1.7 and 61 B.1.177 samples. Because of uncertainty regarding sample handling before arrival at the originating diagnostic laboratory and the unrefrigerated transport, it was not possible to determine whether isolation failures were due to samples containing no infectious particles (due to sample degradation) or for other reasons. Such reasons could include systematic handling differences according to variant type or a difference in virion stability and durability regarding environmental factors such as temperature. Therefore, samples with negative isolation outcome were excluded from analysis. The strong likelihood of many cases of complete sample degradation is evident from the isolation failure of many samples with high pre-inoculation viral load, with the viral load in these cases merely indicating the presence of noninfectious SARS-CoV-2 RNA (fig. S4). Given this context, we were reduced to questioning whether there might be a difference in the range of viral loads that were able to result in isolation between B.1.1.7 and non-B.1.1.7 variants. Such a difference could result from a difference in the ratio of viral RNA to infectious particles produced by the variants, or from a difference other than viral load in the variants. We examined the distribution of pre-inoculation viral loads from isolation-positive samples from both variants for a difference. No statistically significant difference was found, but in the converse, the isolation-positive sample sizes are too low to support the assertion that the distributions do not differ.

### Estimating viral load time course

Each RT-PCR test in our dataset has a date, but no information regarding the suspected date of subject infection or onset of symptoms (if any). Although determining the day of peak viral load for a single person based on a series of dated RT-PCR results would not in general be feasible because of individual variation, data from a large enough set of people would enable the inference of a clear and consistent model of viral load change over time with very few assumptions.

We included a single leading and/or trailing negative RT-PCR result, if dated within 7 days of the closest positive RT-PCR. To produce a model of typical viral load decline on a reasonable single-infection time scale, we excluded subjects whose full time series contains positive RT-PCRs spread over a period exceeding 30 days. Such time series may be attributable to contamination, to later swabbing that picks up residual RNA fragments in tonsillar tissue (*66*), or to re-infection (*67*–*69*), or they may represent atypical infection courses (such as in immunocompromised or severely ill elderly patients) (*70*). We excluded data from subjects with an infection delimited by both an initial and a trailing negative test when there was only a single positive RT-PCR result between them.

We estimated the slopes for a model of linear increase and then decline of log_10_(viral load). To compensate for the absence of information regarding time of infection, we also estimated the number of days from infection to the first positive test for each participant, so as to position the observed time series relative to the day of peak viral load. The analysis was implemented in two ways. Initially, simulated annealing was used to find an optimized fit of the parameters, minimizing a least-squares error function. Second, a Bayesian hierarchical model estimated subject-specific time courses, imputed the viral load assigned to each initial or trailing negative test, and captured effects of age, gender, clinical status, and RT-PCR system with model parameters. We tested both methods on data subsets ranging from subjects with at least three to at least nine RT-PCR results. The two methods produced results that were in generally good agreement (table S5). The finer-grained Bayesian approach appears more sensitive than the simulated annealing; its results, for subjects with at least three RT-PCR results, are those described in the main text.

*Simulated annealing approach*: A simulated annealing optimization algorithm (*71*) was used to adjust the time series for each subject slightly earlier or later in time, by amounts drawn from a normal distribution with mean 0.0 and standard deviation 0.1 days. The error function was the sum of squares of distances of each viral load from a viral load decline line whose slope was also adjusted as part of the annealing process. In the error calculation, negative test results were assigned a viral load of 2.0, in accordance with our SARS-CoV-2 assay limit of detection and sample dilution (*19*). The initial slope of the decline line was set to –2.0 and was varied using *N*(0, 0.01). A second, optional, increase line initialized with a slope of 2.0, adjusted using an *N*(0, 0.01) random variable, was included in the error computation if the day of a RT-PCR test was moved earlier than day zero (the modeled day of peak viral load). The height of the intercept (i.e., the estimated peak viral load) between the increase line (if any) and the decline line was also allowed to vary randomly [starting value 10.0, varied using *N*(0, 0.1)]. The full time series for each subject was initialized with the first positive result positioned at day 2 + *N*(0.0, 0.5) after peak viral load. The random-move step of the simulated annealing modified either of the two slopes or the intercept, each with probability 0.01, otherwise (with probability 0.97) one subject’s time series was randomly chosen to be adjusted earlier or later in time. After the simulated annealing stage, each time series was adjusted to an improved fit (when possible) based on the optimized increase and decline lines. Linear regression lines were then fitted through the results occurring before and after the peak viral load (*x* = 0) and compared to the lines with slopes optimized by the simulated annealing alone. This final step helped to fine-tune the simulated annealing, in particular sometimes placing a time series much earlier or much later in time after it had stochastically moved initially in a direction that later (when the increase and decline line slopes had converged) proved to be suboptimal. The slopes of the lines fitted via linear regression after this final step were in all cases very similar (generally ±0.1) to those produced by the initial simulated annealing step. The final adjustments can be regarded as a last step in the optimization, using a steepest-descent movement operator instead of an uninformed random one. A representative optimization run for subjects with at least three RT-PCR results is shown in fig. S12.

*Bayesian approach*: The Bayesian analysis of viral load time course implements the same basic model, and additionally estimates associations of model parameters with covariates age, gender, B.1.1.7 status, and clinical status, estimates subject-level parameters (slope of log_10_ viral load increase, peak viral load, slope of log_10_ viral load decrease) as random effects, and accounts for effects of PCR system and test center types with random effects. To estimate the number of days from infection to the first test (henceforth “shift”), we constrained the possible shift values from –10 to 20 days and used a uniform prior on the support. In contrast to the other subject-level parameters, we estimated subject-level shifts independently (i.e., without a hierarchical structure). Figure S7 shows the placement in time of individual viral loads after shifting for subjects with RT-PCR results from at least 3 days. Model parameters changed gradually when subsets of subjects with an increasing minimum number of RT-PCR results, from three to nine, were examined (fig. S11 and table S5). The viral load assigned to negative test results (which may include viral loads below the level of detection) is estimated with a uniform prior on the support from –Inf to 3 (see also the caption of fig. S7). Using prior predictive simulations, we specified (weakly) informative priors for this analysis. This analysis was implemented in Stan (*72*), as described in (*97*).

Checking convergence of the model parameters showed that although 99.3% of all parameters converged with an R-hat value below 1.1, some subject-level parameters of 118 subjects (among 4344 subjects with at least three RT-PCR results) showed R-hat values between 1.1 and 1.74. Inspection of these parameters showed that these convergence difficulties were due to observed time courses that could arguably be placed equally well at the beginning or a later stage of the infection. Figure S16 shows a set of 81 randomly selected posterior predictions, to give an impression of time-series placement; fig. S17 shows the 49 participants with the parameters with the highest R-hat values. Although the high R-hat values could be removed by using a mixture approach to model shift for these participants, in light of their low frequency we retained the simpler model to avoid additional complexity. Alternatively, constraining the shift parameter to negative numbers would also improve R-hat values for these subjects, at the cost of the additional assumption that infections are generally not detected weeks after infection.

*Sensitivity analysis*: In addition to examining the viral load time series of subjects with RT-PCR results on at least 3 days, we tested both approaches on data from subjects with results from a minimum of 4 to 9 days. Given the degree of temporal viral load variation seen in other studies (*18*–*20*, *35*, *41*, *46*, *63*, *73*, *74*) and in our own data, our expectation was that a relatively high minimum number of results might be required before reliable parameter estimates with small variance would be obtained, but this proved not to be the case. The simulated annealing approach was tested with a wide range of initial slopes and intercept heights as well as seven different methods for the initial placement of time series. In general, maximum viral load and decline slopes were robust to data subset and initial time-series location, although there was variation in the length of the time to peak viral load, depending on how early in time the time series were initially positioned, the initial slopes of the increase and decrease lines and height of the maximum viral load. This is as expected, as the settings of these parameters can be used to bias the probability that a time series is initially positioned early or late in time and how difficult it is for it to subsequently move to the other side of the peak viral load at day zero. Table S5 shows parameter values for both approaches on the various data subsets.

*Onset of shedding*: We define the onset of shedding as the time point at which the increasing viral load crosses zero of the log_10_ *y* axis—that is, when just one viral particle was estimated to be present. Because the estimated time of infection depends on the estimated peak viral load and the slope with which viral load increases, the data should optimally include multiple pre-peak viral load test results for each individual. If, as in the current dataset, only a subset of subjects have test results from pre-peak viral load, a hierarchical modeling approach still allows calculating subject-level estimates. Intuitively, this approach uses data from all subjects to calculate an average slope parameter for increasing viral load. In addition, it models subject-level parameters as varying around the group-level parameter. To further refine the estimation of slope parameters, the model also uses the age (see fig. S10), gender, and clinical status as covariates. Because negative test results could be false negatives, viral loads for these tests are imputed (with an upper bound of 3). Subject-level peak viral load and declining slope are modeled with the same approach. More generally, using a hierarchical model and shrinkage priors for the effects of covariates results in more accurate predictions in terms of expected squared error (*75*) compared to analyzing each subject in isolation, but the overall improvement introduces a slight bias toward the group mean, resulting in an underestimation of the true variability of subject-level parameters. This is especially the case if, as in the current dataset, subject-level data are sparse.

*Onset of symptoms*: The 317 onset-of-symptoms dates for hospitalized patients were collected as part of the Pa-COVID-19 study, a prospective observational cohort study at Charité–Universitätsmedizin Berlin (*76*, *77*), approved by the local ethics committee (EA2/066/20), conducted according to the Declaration of Helsinki and Good Clinical Practice principles (ICH 1996), and registered in the German and WHO international clinical trials registry (DRKS00021688).

### Software

The following Python (version 3.8.2) software packages were used in the data analysis and in the production of figures: Scipy (version 1.4.1) (*78*), pandas (version 1.0.3) (*79*), statsmodels (version 0.11.1) (*80*), matplotlib (version 3.2.1) (*81*), numpy (1.18.3) (*82*), seaborn_sinaplot (*83*), simanneal (version 0.5.0) (*71*), and seaborn (version 0.10.1) (*84*). Sequence analysis used Bowtie2 (2.4.1) (*85*), bcftools and samtools (1.9) (*86*, *87*), Geneious Prime (2021.0.3) (*88*), ivar (1.2.2) (*89*), and MAFFT (4.475) (*90*). Analyses in R (4.0.2) (*60*) were conducted using the following main packages: brms (2.13.9) (*58*, *59*), rstanarm (2.21.1) (*91*), rstan (2.21.2) (*92*), data.table (1.13.3) (*93*), and ggplot2 (3.3.2) (*94*). Bayesian analysis in R was based on Stan (2.25) (*72*). Parallel execution was performed with GNU Parallel [20201122 (‘Biden’) (*95*)].

### Data curation and anonymization

Research clearance for the use of routine data from anonymized subjects is provided under paragraph 25 of the Berlin *Landeskrankenhausgesetz*. All data are anonymized before processing to ensure that it is not possible to infer patient identity from any processing result. All patient information is securely combined into a token that is then replaced with a value from a strong one-way hash function prior to the distribution of data for analysis. Viral loads are calculated from RT-PCR cycle threshold values that have only one decimal place of precision.

## Supplementary Materials

science.sciencemag.org/content/373/6551/eabi5273/suppl/DC1
Supplementary Text Figs. S1 to S17 Tables S1 to S5 References (*98*–*102*) MDAR Reproducibility Checklist
